# Validation of Reliable Reference Genes for RT-qPCR Studies of Target Gene Expression in *Colletotrichum camelliae* During Spore Germination and Mycelial Growth and Interaction With Host Plants

**DOI:** 10.3389/fmicb.2019.02055

**Published:** 2019-09-04

**Authors:** Shengnan He, Tai An, Runa A, Shouan Liu

**Affiliations:** Laboratory of Molecular Plant Pathology, College of Plant Science, Jilin University, Changchun, China

**Keywords:** reference genes, tea plant, *Colletotrichum camelliae*, RT-qPCR, gene expression

## Abstract

The tea plant [*Camellia sinensis* (L.) O. Kuntze] is one of the most important leaf crops, and it is widely used for the production of non-alcoholic beverages worldwide. Tea also has a long history of medicinal use. *Colletotrichum camelliae* Massee is one of the dominant fungal pathogens that infects tea leaves and causes severe tea anthracnose disease. To analyze the molecular biology of *C. camelliae*, the quantification of pathogen gene expression by the RT-qPCR method is necessary. Reliable RT-qPCR results require the use of stable reference genes for data normalization. However, suitable reference genes have not been reported in *C. camelliae* thus far. In this study, 12 candidate genes (i.e., *CcSPAC6B12.04c*, *CcWDR83*, *Cchp11*, *Ccnew1*, *CcHplo*, *CcRNF5*, *CcHpcob*, *CcfaeB-2*, *CcYER010C*, *CcRNM1*, *CcUP18*, and *CcACT*) were isolated from *C. camelliae* and assessed as potential reference genes. The expression stability of these genes in *C. camelliae* during spore germination and mycelial growth and interaction with host plants was first evaluated using several statistical algorithms, such as geNorm, NormFinder, and Bestkeeper. A web-based analysis program, Refinder, was then used to find the most suitable reference genes. Our results indicated that *Cenew1*, *CcHplo*, and *CcSPAC6B12.04c* were the most stable reference genes in *C. camelliae* under all conditions. Our work provided the most suitable reference genes for future studies performed to quantify the target gene expression levels of *C. camelliae.*

## Introduction

*Colletotrichum* includes a wide range of fungal pathogens that cause serious diseases in various plants in tropical, subtropical, and temperate regions (Kubo, 2012; Yan et al., 2018). Their economic impacts have led to extensive studies on diverse aspects of fungal biology, including fungi–plant interactions, genomics and genetics, the cell biology of pathogen infection and colonization, and fungal virulence factors (De Silva et al., 2017; Villa-Rivera et al., 2017; Yan et al., 2018). Some species have been used as models for studying infection strategies and host-parasite interactions (De Silva et al., 2017; Yan et al., 2018). *Colletotrichum camelliae* is one of the dominant fungal pathogens that infects tea plants (Wang Y. et al., 2016; Lu et al., 2018). *C. camelliae* can damage tea leaves and cause several tea diseases, such as tea anthracnose, tea leaf blight, and tea brown blight (Chen and Chen, 1990; Liu F. et al., 2015; Wang L. et al., 2016; Wang Y. et al., 2016; Lu et al., 2018).

The quantification of functional gene expression levels is one of the most important aspects in the systematic study of gene transcription and regulation (Marcial-Quino et al., 2016). The reverse transcription quantitative real-time polymerase chain reaction (RT-qPCR) method is frequently used to quantify target gene expression levels (Derveaux et al., 2010; Borowski et al., 2014; Mcintosh et al., 2016). The RT-qPCR method is simple, reproducible, highly sensitive, and practical in detecting gene transcription (Klein, 2002; Hao et al., 2014). However, reliable RT-qPCR results require suitable reference genes for data analysis. The use of inadequate reference genes may result in incorrect expression data (Amil-Ruiz et al., 2013; Galli et al., 2015). Thus, the suitable reference genes in *C. camelliae* should be constantly expressed among the samples, and their expression is assumed to be unaffected by different experimental conditions (Bustin, 2002; Galli et al., 2015).

*ACT*, *TUB*, *GAPDH*, and *18SRNA* are often used as reference genes because of their functions in basic cellular processes, cell structure maintenance, or primary metabolism (Galli et al., 2015). Reports have shown that several traditional reference genes were used in *Colletotrichum* spp.; for example, the *ACT* gene was used in *C. higginsianum* not only to quantify fungal growth but also to normalize the expression of the MFS transporter gene *ChMFS1* (Narusaka et al., 2009; Liu et al., 2017). In *C. acutatum* and *C. gloeosporioides*, the β*-TUB* gene was used to normalize the expression of two ABC genes, *CaABC1* and *CgABCF2* (Kim et al., 2014; Zhou et al., 2017). In *C. lindemuthianum* and *C. coccodes*, two conserved genes, *GAPDH* and *18SRNA*, were also used as reference genes (Ben-Daniel et al., 2012; Pereira et al., 2013; Galli et al., 2015). However, no suitable reference genes have been reported in *C. camelliae* for RT-qPCR analysis, although stably expressed referenced genes are believed to occur in *C. camelliae*, even under different experimental conditions. Thus, suitable reference genes in *C. camelliae* should be identified.

In the present study, we evaluated the stability of 12 candidate genes to identify the most suitable reference genes for transcript normalization in *C. camelliae* during spore germination and mycelial growth and interaction with hosts. To evaluate the efficacy of selected reference genes, we investigated the expression of a target gene involved with an ABC transporter during *C. camelliae* spore germination and mycelial growth and its interaction with tea plants. To our knowledge, this is the first analysis of the expression stability of suitable reference genes in *C. camelliae*.

## Materials and Methods

### Plants, Pathogen Materials, and Treatments

Tea plant *Camellia sinensis* cultivar Longjing 43 (LJ43) was used for all assays. Two-year-old tea were grown in a microbe-free climate chamber under 12-h light/12-h dark conditions at 25°C and 60–80% relative humidity before inoculation. For fungal inoculation, mature leaves of 2-year-old LJ43 were collected randomly. The *C. camelliae* isolate CCA was isolated from a diseased garden in Fancun, which is located in Hangzhou, China. The fungal isolate was cultivated on PDA plates at 22°C in a climate chamber (12-h light/12-h dark) for 10 days. *C. camelliae* spores were then collected, washed, and frozen at −80°C in 0.8% NaCl with a concentration of 10^8^ spores mL^–1^ as previously indicated (Liu S. et al., 2015).

For the inoculation of tea plants, spores were diluted in ddH_2_O with a final concentration of 10^6^ spore mL^–1^. Six to eight droplets (20 μL for one droplet) of diluted spores were applied to each single detached tea leaf. The leaves were wounded with a narrow razor blade before inoculation. In each treatment, at least 40 mature leaves were randomly selected from more than 20 tea plants. For the control, spores were incubated in ddH_2_O. Infection was carried out on a bench at room temperature. After infection for different times (e.g., 12, 14, or 24 h), the fungi were recovered from tea leaves and then frozen at −80°C for RNA assays. Three independent biological replicates were performed.

For the effects of tea catechins (Aladdin, China) on *C. camelliae* gene expression, the fungus was incubated on PDA solid media (Lu et al., 2018). The compound was dissolved in ddH_2_O and then mixed with sterile melted PDA medium to obtain a final concentration of 0.25 mg mL^–1^. The PDA medium was then poured into 9.0 cm diameter Petri plates for the inoculation with 0.8 cm disks of *C. camelliae* CCA. Each treatment was performed in 3 replicates. The PDA plates containing ddH_2_O (without any tea catechins) were used as the control. Fresh spores of *C. camelliae* CCA were also used as a 0-h control. The mycelia were harvested at 3 and 6 days, respectively. The fresh spores and mycelia were frozen at −80°C and used for RNA assays.

### Total RNA Extraction and Reverse Transcription

Approximately 0.1 mg fungal fresh mycelia or 10^6^ spores were used for RNA extraction. The samples were first frozen in liquid nitrogen and homogenized using a Tissue Lyser (Qiagen, Hilden, Germany) for 2 × 30 sec at 30 strokes/sec. RNA was then extracted using 1.0 mL TRIzol^®^ Reagent (Life Technologies, Foster City, CA, United States) according to the manufacturer’s instructions. Finally, the total RNA was dissolved in nuclease-free water. The purity and concentration of the isolated RNA were estimated by a Nanodrop ND-1000 spectrophotometer (Nanodrop Technologies, Wilmington, DE, United States). RNA with an A260/A280 ratio of 1.8–2.0 was used for cDNA synthesis. The quality and integrity of the purified RNA templates was further confirmed by agarose gel electrophoresis. cDNA was synthesized from 1.0 μg of total RNA using the PrimeScript^TM^ RT reagent kit with gDNA Eraser (Takara, Dalian, China) to remove the genomic DNA contamination.

### Selection of Candidate Reference Genes and Primer Design

*CcACT* was chosen as a PCR reference gene in the present study according to previous reports (Wang Y. et al., 2016; Ning et al., 2018). Eleven candidate reference genes were chosen from the transcriptomic data of *C. camelliae* during the infection of tea plants (Supplementary Table S1). The open reading frame (ORF) sequences of 11 candidate reference genes from *C. camelliae* were first cloned using 2x Primer Star mix (Takara, Dalian, China) as the polymerase. The purified PCR products were ligated into the pEASY^®^-Blunt simple cloning vector (TranGen, Beijing, China) and then transformed into *Escherichia coli*. The bacterial liquids were sequenced by Comate Bioscience (Comate, Changchun, China). A bioinformatics analysis of the reference gene was performed by BLAST^1^. The RT-qPCR primers for all candidate genes were designed by Primer-BLAST^2^ and are presented in Table 1.

**TABLE 1:** Description and characterization of the candidate reference genes for RT-qPCR.

**Reference genes**	***Colletotrichum* ortholog**	***Colletotrichum* locus description**	**Forward/reverse primer sequence (5′–3′)**	**Amplicon size (bp)**	**RT-qPCR efficiency (%)**	**Tm (°C)**
*CcRNM1*	EQB58236	Ran-interacting Mog1 protein	ACGGGCGTTATTTGGACC/CACACAGTGAGGGTAGCGAAT	148	99.91–99.96	85.2
*CcSPAC6B12.04c*	ELA24928	Kynurenine aminotransferase	CATTGCCGAGGACTACATCC/TTCTTCAACCCACGCAGC	86	99.91	85.2
*CcHpcob*	EQB50494	Hypothetical protein CGLO_10066	ACGAGCCTGTTCAGAATGCTA/GACTTGCTATCTTGGACGGGT	87	99.98–99.99	84.9
*Ccnew1*	EQB44976	Hypothetical protein CGLO_16211	CGTGGCTTTGAAATCAGACC/CCATCTCGTGACTAGGAGCAA	107	99.99–100	84.3
*CcHplo*	EQB45128	Hypothetical protein CGLO_16041	ACATAGCATCGCATCCCG/TTCCTCAGGGTCGAACTCC	124	99.98–99.99	86.4
*CcfaeB-2*	EQB48532	Hypothetical protein CGLO_12218	GACCTGTCGGCTTGTCTGAC/AGGTTGTGGACATTCGCATC	95	99.49–99.94	86.4
*CcWDR83*	EQB49172	Hypothetical protein CGLO_11518	TAATCGCTGGAGACGAGTTGA/TCTTGGGTTCATAGCCTTCG	140	99.99	87.3
*Cchp11*	EQB55695	Hypothetical protein CGLO_04353	TTTCGCAAGCCCTCTTTG/CAAGCCTTTGGTTTCCCTG	82	99.91–99.96	82.5
*CcRNF5*	EQB48134	Hypothetical protein CGLO_12656	TGACGACGACATCTTTGGC/ACGCATGTAAACGCGGAC	158	99.98–99.99	85.8
*CcYER010C*	EQB57386	DlpA domain-containing protein	GATCAACCCTGGCGATATTCT/TCCGCAACTCAACAAACATCT	133	99.94–99.99	85.8
*CcUP18*	ELA35911	Hypothetical protein CGGC5_4557	GGATCGAGAGAGGGACTTGC/CTCTGTCGTCGTCCTTGTCC	94	99.9–99.99	86.1
*CcACT*	KJ954363	Actin	GTTTCGCCGGTGACGATG/CTGGCCCATACCAATCATGA	78	99.71–99.82	86.4

### Quantitative Real-Time PCR

For qPCR analysis, approximately 20 ng of cDNA was mixed with 0.2 mM gene-specific primers and SYBR Green Supermix in a total volume of 10 μL. The qPCR was performed using a LightCycler^®^ 480 system (Roche) according to the manufacturer’s instructions. The PCR program consisted of a preliminary step of 1 min at 95°C followed by 40 cycles at 95°C for 15 s and at 60°C for 34 s. No-template and no-RT controls for each primer pair were included. Each qPCR was performed in triplicate, and each experiment was independently repeated for three times. Standard curves were drawn to determine the amplification efficiency (E) and correlation coefficient (*R*^2^) of the diluted series on the basis of the 10-fold diluted cDNA series (Wu et al., 2016). We used the following equation to calculate the qPCR efficiency: E = (10^{−1/slope}^−1) × 100%.

### Validation of Reference Genes

The expression of the 12 candidate genes was first evaluated according to the quantification cycle (Cq) value. Determination of the expression stability of the genes was then performed with three statistical algorithms (Bestkeeper, NormFinder, and geNorm) for the evaluation and selection of reference genes (Vandesompele et al., 2002; Andersen et al., 2004; Pfaffl et al., 2004; Marcial-Quino et al., 2016). The Cq values obtained for each of the analyzed genes were used to monitor the stability of the genes with these three methods, thus identifying the best reference genes for the normalization of data in RT-qPCR analyses (Marcial-Quino et al., 2016). Finally, Refinder was used to comprehensively evaluate and rank the reference genes from experimental data (Xie et al., 2011).

For the validation and quantification of the target gene (*CcABC8*) expression after pathogen infection, the 2^–ΔΔCT^ method was used (Livak and Schmittgen, 2001). Statistical analyses were performed using a Student’s homoscedastic two-tailed *t-*test. Statistical significance is considered at ^∗^*P* < 0.05 and ^∗∗^*P* < 0.01. Three replicates of three independent experiments were performed. The qPCR primers for *CcABC8* included CcABC8S (5′-TCCCTCCTCCTGACTCTCCT-3′) and CcABC8A (5′-TGGATCAATGTTGTCACGGA-3′).

## Results

### Isolation and Characterization of Candidate Reference Genes

The candidate reference genes were identified from the *C. camelliae* transcriptome by a homology analysis with *Colletotrichum* spp. (Supplementary Table S1). The transcripts of these genes were slightly changed during *C. camelliae* spore germination and during its interaction with host plants, thereby we chose them for further analysis. The ORF sequence of each gene was cloned from *C. camelliae* CCA based on the transcriptomic data (Supplementary Figure S1 and Supplementary Table S2). Protein-protein BLAST analysis indicated that two genes had the highest homology ( 89.5 and 98.9%) with *C. fructicola* Nara gc5, while others had the highest homology with *C. gloeosporioides* Cg-14 (from 82.3 to 99%) (Alkan et al., 2013; Gan et al., 2013). Among these candidate reference genes, one was similar to the ran-interacting Mog1 protein and named *CcRNM1*, a second gene was similar to kynurenine aminotransferase and named *CcSPAC6B12.04c*, a third gene was a DlpA domain-containing protein and referred to as *CcYER010*, and other genes were annotated as hypothetical proteins and named *CcHpcob*, *Ccnew1*, *CcHplo*, *CcfaeB-2*, *CcWDR83*, *Cchp11*, *CcRNF5*, and *CcUP18*.

The bioinformatics results for the amino acid sequence information are shown in Supplementary Table S2. The total amino acid number for the candidate reference gene was from 78 for Ccnew1 to 876 for CcHpcob; therefore, the lowest predicted molecular weight (MW) was 8.6 kDa for Ccnew1, and the highest MW was 100.5 kDa for CcHpcob. Most of the proteins had MWs ranging from 20 to 65 kDa. The predicted isoelectric point (pI) was from 4.3 for CcRNM1 to 11.0 for CcUP18, and many of proteins had pI values from 4.5 to 7.5.

### Determination of Primer Specificity and Efficiency

Based on the candidate reference genes observed, 12 pairs of primers were designed (Table 1). A traditional reference gene, *CcACT*, was also isolated and included. The cDNA was synthesized from RNA obtained from organisms isolated at 12 h after infection of the plant cultivar Longjing 43. *C. camelliae* spores inoculated with ddH_2_O were used as a non-plant inoculation control (CK).

**FIGURE 1:**
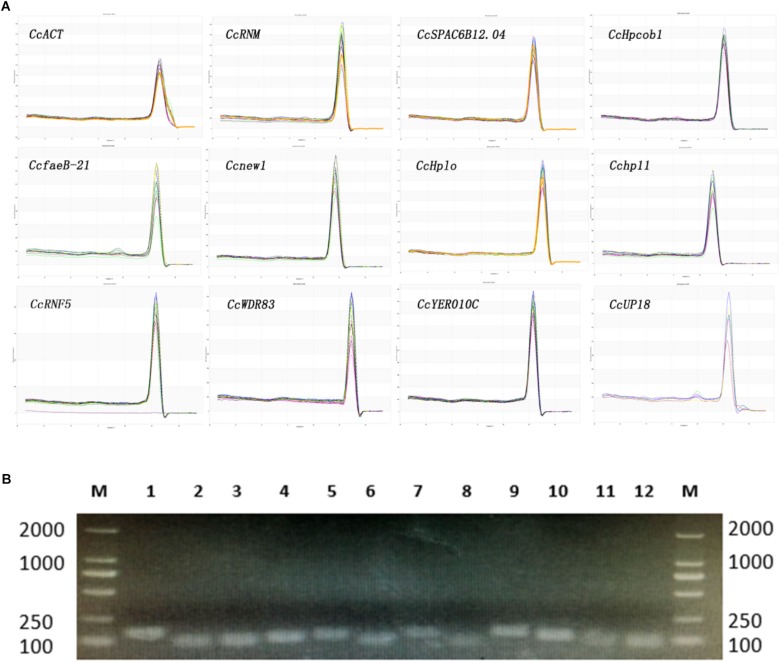
Confirmation of the primer specificity and amplicon size. **(A)** Melting curve analysis of 12 candidate reference genes. All RT-qPCR products had a single melting curve indicating the breakdown of only one PCR product. **(B)** Amplification results for 12 candidate genes using a *C. camelliae* cDNA template. M: DL2000 DNA marker. 1–12. *CcRNM1*, *CcSPAC6B12.04c*, *CcHpcob*, *Ccnew1*, *CcHplo*, *CcfaeB-2*, *CcWDR83*, *Cchp11*, *CcRNF5*, *CcYER010C*, *CcUP18*, and *CcACT*.

The melting curves observed from the PCR amplification products for each of the genes showed a single distinct sharp peak (Figure 1A). The specificity of the primers for all genes was further tested in 2.0% agarose gels. For all the candidate reference genes, a single PCR amplification band of the expected size was observed (Figure 1B), indicating that primer dimers and non-specific amplified products were not generated (Marcial-Quino et al., 2016).

**FIGURE 2:**
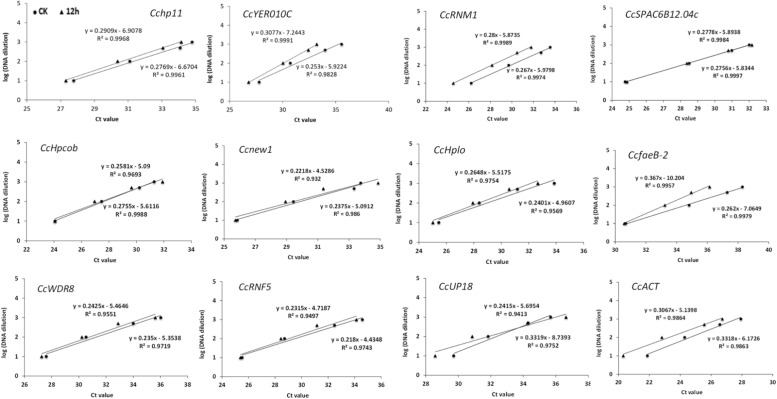
Validation of primers for RT-qPCR quantification of the tea pathogen. The primer efficiency for the RT-qPCR quantification of the gene was determined using a serial dilution of cDNA templates from *C. camelliae*. The respective correlation coefficients (*R*^2^) are indicated.

In a second experiment, the efficiency of the primers was detected using a 10-fold dilution series of cDNA from *C. camelliae* CCA (CK and 12 h). For all of the above cDNA samples, the PCR amplification efficiencies for the candidate genes varied from 99.49% for *CcfaeB-2* to 100.00% for *Ccnew1* (Table 1). The primers yielded linear amplification on a range of cDNA concentrations and the correlation coefficient *R*^2^ were from 93.2% for *Ccnew1* to 99.97% for *CcSPAC6B12.04c* (Figure 2). For the gene primers, such as *CcSPAC6B12.04c*, *Cchp11*, *CcRNM1*, and *CcfaeB-2*, *R*^2^ was over 99%, indicating that these primer pairs were well-suited for amplification of the gene even when using very low cDNA input.

### Expression Profiles of Candidate Reference Genes

A reliable reference gene should present a constant expression level among different samples or different conditions (Galli et al., 2015); thus, we next checked the Cq values to evaluate the expression of these candidate reference genes. Total RNA was extracted from *C. camelliae* CCA fresh spores (un-germinated spores, CcFS0h), *C. camelliae* grown on PDA plates for 3 days (mycelium, CcPM3d) and 6 days (mycelium, CcPM6d), *C. camelliae* grown on PDA plates with tea catechins for 3 days (mycelium, CcPCM3d) and 6 days (mycelium, CcMPC6d), *C. camelliae* incubated on tea plant LJ43 for 14 h (CcTP14h) and *C. camelliae* spores incubated with ddH_2_O for 14 h (control, spores germinated in ddH_2_O, CcGe14h). Total RNA was reverse transcribed into cDNA and then used for RT-qPCR.

**FIGURE 3:**
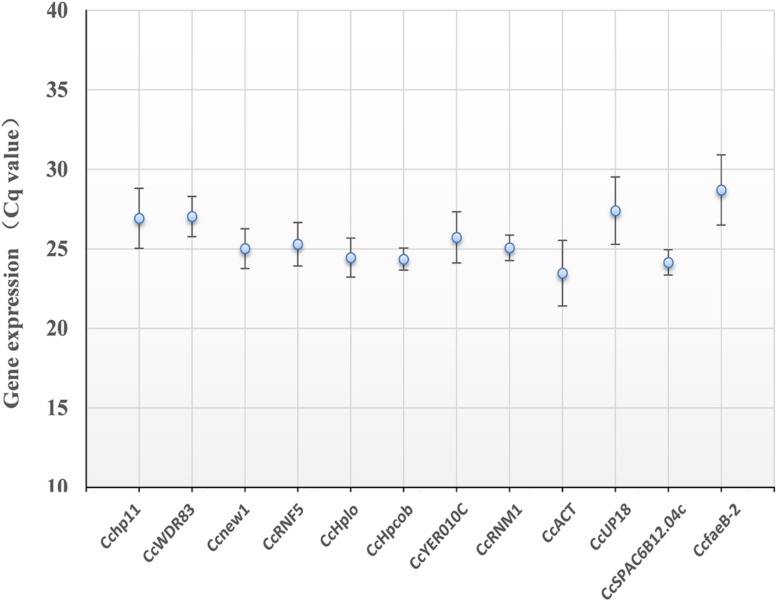
Average cycle threshold (Cq) values for 12 candidate reference genes. The filled dot symbol indicates the mean Cq values. The bars indicate standard deviation.

As shown in Figure 3, the 12 candidate reference genes showed a narrow Cq range among all experimental series. The Cq values ranged from 23.5 to 28.7. Transcription of *CcACT* showed the most abundant level, while the gene *CcfaeB-2* was the least abundant transcript. It indicated that each reference gene had varied expression ranges in all studied samples (Hao et al., 2014). *CcHpcob* had the lowest variation in expression across the studied reference genes, while *CcfaeB-2* showed the highest variation in expression (Figure 3).

### Expression Stability of the Candidate Genes

First, to calculate the reference gene expression stability, geNorm software was used (Vandesompele et al., 2002). According to geNorm, the candidate gene that had the lowest value was considered the most stable gene (Zhao et al., 2019). As shown in Table 2, *Ccnew1*, *CcfaeB-2*, *CcWDR83*, *CcHpcob*, and *CcHplo* were the top five stable candidate reference genes during *C. camelliae* spore germination and its interaction with hosts (group 1: CcFS, CcGe, and CcTP), while *Ccnew1*, *CcfaeB-2*, *CcSPAC6B12.04c*, *CcHplo*, and *CcYER010C* were the top five genes during mycelial growth (group 2: CcM). Three genes, *Ccnew1*, *CcfaeB-2* and *CcHplo*, were detected in both groups, indicating that these genes were stable under each condition. Furthermore, in a combined group analyzed by geNorm software, the top five stable candidate reference genes were *Ccnew1*, *CcHplo*, *CcWDR83*, *CcSPAC6B12.04c*, and *CcHpcob*. This finding indicates that these genes are relatively stable during spore germination and mycelial growth and infection processes.

**TABLE 2:** Gene expression stability ranked by geNorm, NormFinder, and BestKeeper software programs.

**Group**	**Rank**	**geNorm**		**NormFinder**		**BestKeeper**		
		**Gene**	**Stability**	**Gene**	**Stability**	**Gene**	**SD**	**CV**
Spore germination and interaction with hosts (group 1)	1	*Ccnew1 | CcfaeB-2*	0.299	*Ccnew1*	0.269	*CcSPAC6B12.04c*	0.37	1.49
	2			*CcWDR83*	0.315	*CcHpcob*	0.53	2.18
	3	*CcWDR83*	0.338	*CcfaeB-2*	0.318	*CcHplo*	0.55	2.15
	4	*CcHpcob*	0.374	*CcHpcob*	0.356	*Ccnew1*	0.58	2.23
	5	*CcHplo*	0.404	*CcHplo*	0.437	*CcYER010C*	0.6	2.21
	6	*CcACT*	0.549	*CcYER010C*	0.581	*CcfaeB-2*	0.61	1.96
	7	*CcYER010C*	0.629	*CcACT*	0.672	*CcACT*	0.63	2.98
	8	*CcUP18*	0.689	*CcUP18*	0.726	*CcRNM1*	0.63	2.51
	9	*CcRNM1*	0.754	*Cchp11*	0.904	*CcWDR83*	0.73	2.6
	10	*CcSPAC6B12.04c*	0.803	*CcRNM1*	0.937	*CcRNF5*	0.96	3.67
	11	*Cchp11*	0.856	*CcSPAC6B12.04c*	0.964	*CcUP18*	0.97	3.28
	12	*CcRNF5*	0.94	*CcRNF5*	1.231	*Cchp11*	1.19	4.2
Mycelial growth (group 2)	1	*Ccnew1 | CcfaeB-2*	0.144	*Ccnew1*	0.099	*CcACT*	0.55	2.2
	2			*CcfaeB-2*	0.185	*Ccnew1*	0.56	2.3
	3	*CcSPAC6B12.04c*	0.225	*CcHplo*	0.205	*CcfaeB-2*	0.56	2.07
	4	*CcHplo*	0.28	*CcSPAC6B12.04c*	0.292	*CcSPAC6B12.04c*	0.58	2.42
	5	*CcYER010C*	0.304	*CcRNM1*	0.326	*CcHpcob*	0.58	2.39
	6	*CcRNF5*	0.333	*CcRNF5*	0.338	*CcHplo*	0.61	2.6
	7	*CcRNM1*	0.38	*CcYER010C*	0.343	*CcRNM1*	0.62	2.48
	8	*CcHpcob*	0.426	*CcHpcob*	0.483	*CcRNF5*	0.63	2.57
	9	*CcACT*	0.472	*CcACT*	0.602	*CcYER010C*	0.69	2.8
	10	*CcUP18*	0.526	*CcUP18*	0.694	*CcWDR83*	0.7	2.67
	11	*CcWDR83*	0.593	*CcWDR83*	0.813	*CcUP18*	0.71	2.74
	12	*Cchp11*	0.652	*Cchp11*	0.844	*Cchp11*	1.09	4.21
Total (combined group)	1	*Ccnew1 | CcHplo*	0.288	*Ccnew1*	0.115	*CcHpcob*	0.56	2.31
	2			*CcHplo*	0.242	*CcSPAC6B12.04c*	0.6	2.5
	3	*CcWDR83*	0.579	*CcWDR83*	0.545	*CcRNM1*	0.63	2.52
	4	*CcSPAC6B12.04c*	0.771	*CcSPAC6B12.04c*	0.673	*Ccnew1*	0.91	3.61
	5	*CcHpcob*	0.86	*CcRNF5*	0.746	*CcHplo*	0.99	4.0
	6	*CcRNM1*	0.896	*CcYER010C*	0.794	*CcRNF5*	1.1	4.48
	7	*CcRNF5*	0.95	*CcHpcob*	0.795	*CcWDR83*	1.12	4.46
	8	*CcYER010C*	0.991	*CcRNM1*	0.844	*CcYER010C*	1.45	5.64
	9	*Cchp11*	1.045	*Cchp11*	1.009	*Cchp11*	1.6	5.94
	10	*CcfaeB-2*	1.129	*CcUP18*	1.423	*CcUP18*	1.89	6.89
	11	*CcUP18*	1.18	*CcfaeB-2*	1.442	*CcACT*	1.92	8.18
	12	*CcACT*	1.5	*CcACT*	3.034	*CcfaeB-2*	2.08	7.26

*SD, standard deviation; CV, coefficient of variation.*

NormFinder software is based on a mathematical model of separate analyses of sample subgroups and the estimation of both intra- and intergroup expression variations (Andersen et al., 2004; Wu et al., 2016). Genes with stable expression were indicated by low average expression stability values (Wu et al., 2016). Based on NormFinder analysis, *Ccnew1*, *CcWDR83*, *CcfaeB-2*, *CcHpcob*, and *CcHplo* were the top five stable candidate reference genes in group 1, which were the same as the genes identified by geNorm software (Table 2). In group 2, the top five stable genes were *Ccnew1*, *CcfaeB-2*, *CcHplo*, *CcSPAC6B12.04c*, and *CcRNM1*. Interestingly, *Ccnew1*, *CcfaeB-2*, and *CcHplo* were also observed in both groups. This result was the same as that found by the geNorm analysis and further confirmed that these genes were stable under each condition. In the combined group, the top five stable genes were *Ccnew1*, *CcHplo*, *CcWDR83*, *CcSPAC6B12.04c*, and *CcRNF5*. Based on NormFinder, these genes are relatively stable under all conditions.

Bestkeeper, which calculates the CP standard deviation (SD) and the coefficient of variance (CV) for each gene, was additionally used (Pfaffl et al., 2004; Marcial-Quino et al., 2016). Stable reference genes have a relatively low coefficient of variance and standard deviation (CV ± SD). Genes with SD values < 1 are considered stable and thus are suitable as reference genes (Marcial-Quino et al., 2016). The results of analysis for the 12 reference genes showed markedly stable expression in both group 1 and group 2 samples except *Cchp11* (Table 2). However, under the combined condition, the suitable reference genes observed by Bestkeeper were *CcHpcob*, *CcSPAC6B12.04c*, *CcRNM1*, *Ccnew1*, and *CcHplo*.

**FIGURE 4:**
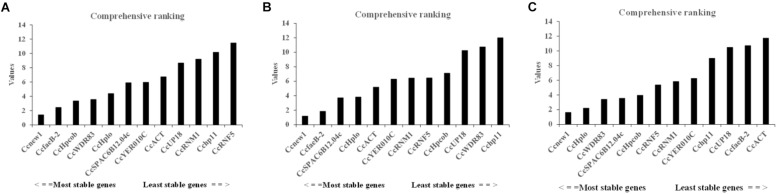
Expression stability of the candidate reference genes in *C. camelliae* as calculated by RefFinder. **(A)** Expression stability of the candidate reference genes during *C. camelliae* spore germination and interaction with tea plants. **(B)** Expression stability of the candidate reference genes during *C. camelliae* mycelial growth. **(C)** Expression stability of the candidate reference genes in *C. camelliae* under the combined condition.

To identify the most suitable reference genes, RefFinder was used to analyze stability of the candidate reference genes. The outcome of four programs (ΔCt, Bestkeeper, geNorm, and NormFinder) were integrated by RefFinder (Xie et al., 2011), and *Ccnew1*, *CcfaeB-2*, *CcHpcob*, and *CcWDR83* appeared to be the most stable reference genes during *C. camelliae* spore germination and its interaction with hosts (group 1), while *Ccnew1*, *CcfaeB-2*, *CcSPAC6B12.04c*, and *CcHplo* were the most stable reference genes during mycelial growth (group 2) (Figures 4A,B). *Ccnew1* and *CcfaeB-2* seemed stable in each group when target gene expression was analyzed separately. When target gene expression was analyzed under both conditions, *Ccnew1*, *CcHplo*, *CcSPAC6B12.04c*, and *CcWDR83* were the most stable reference genes (Figure 4C). However, *CcfaeB-2*, *CcHpcob*, and *CcWDR83* were observed as the least stable reference genes under at least one condition (Figures 4A–C). In conclusion, we considered *Ccnew1*, *CcHplo*, and *CcSPAC6B12.04c* to be the most stable reference genes that could be used to compare and analyze the target gene expression in *C. camelliae* during spore germination, mycelial growth and its interaction with host plants.

### Evaluation of Reference Genes

To evaluate and compare the functional gene expression in *C. camelliae*, the *Cenew1*, *CcSPAC6B12.04c*, *CcHplo*, *Cchp11*, *CcACT*, and *CcUP18* genes were selected as reference genes for RT-qPCR. *Cenew1* was the most stable reference gene under all conditions. *CcSPAC6B12.04c* was the most suitable candidate reference gene even when using a low cDNA input. *CcHplo* was also very stable during mycelial growth and under the combined condition. *CcACT* was the least stable reference gene under the combined condition, while the *Cchp11* and *CcUP18* genes were the least stable reference genes under all conditions. We chose one target gene that may be involved with the ABC transporter (*CcABC8*) to test its expression.

**FIGURE 5:**
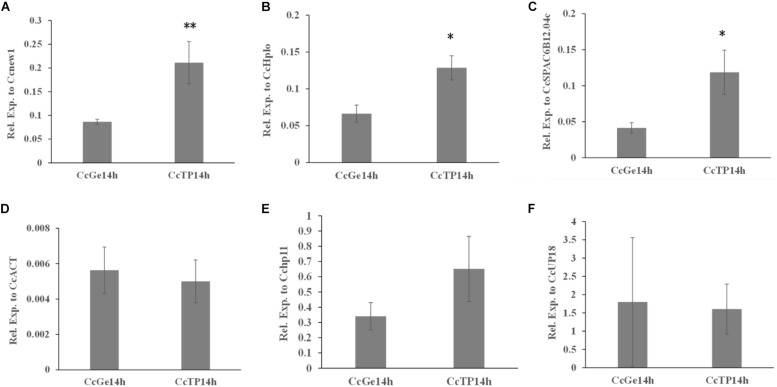
Expression profile of *CcABC8* in *C. camelliae* during spore germination and its interaction with tea plants. The most stable reference genes (*Cenew1*, *CcHplo*, and *CcSPAC6B12.04c*) and the least stable reference genes (*CcACT*, *Cchp11*, and *CcUP18*) were used to normalize the expression data. **(A)** *Cenew1*; **(B)** *CcHplo*; **(C)** *CcSPAC6B12.04c*; **(D)** *CcACT*; **(E)** *Cchp11*; and **(F)** *CcUP18*. The data show the mean expression ± standard deviation calculated from three biological replicates. ^∗^*P* < 0.05; ^∗∗^*P* < 0.01.

As shown in Figures 5D–F, the expression level of *CcABC8* showed no significant differences between *C. camelliae* spore germination (CcGe) and interaction with the tea plant (CcTP) when we used *CcACT*, *Cchp11*, and *CcUP18* as the reference genes. When we used *Cenew1*, *CcSPAC6B12.04c*, or *CcHplo* as the reference genes, we detected significant *CcABC8* gene induction during pathogen interaction with the tea plant (Figures 5A–C).

**FIGURE 6:**
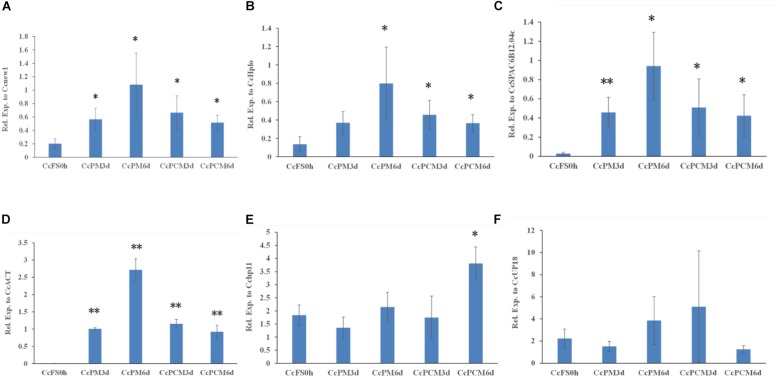
Expression profile of *CcABC8* in *C. camelliae* during mycelial growth. The reference genes (*Cenew1*, *CcHplo*, *CcSPAC6B12.04c*, *CcACT*, *Cchp11*, and *CcUP18*) were used to normalize the expression data. **(A)** *Cenew1*; **(B)** *CcHplo*; **(C)** *CcSPAC6B12.04c*; **(D)** *CcACT*; **(E)** *Cchp11*; and **(F)** *CcUP18*. The data show the mean expression ± standard deviation calculated from three biological replicates. ^∗^*P* < 0.05; ^∗∗^*P* < 0.01.

In addition, the expression of *CcABC8* during *C. camelliae* mycelial growth was also tested. Since the *C. camelliae* spores were incubated on PDA plates at the beginning (CcFS0h) of the experiment, we used CcFS0h as the control. As shown in Figures 6A–D, the expression of *CcABC8* increased during *C. camelliae* growth on PDA plates (CcPM) or PDA plates with catechins (CcPCM) for 3 and 6 days when using *Cenew1*, *CcSPAC6B12.04c*, *CcHplo*, or *CcACT* as reference genes, respectively. This indicates that the gene was induced during mycelial growth. The expression of *CcABC8* was higher after growing on PDA plates for 6 days than 3 days (Figures 6A–D). However, the expression of *CcABC8* was not significantly increased in fungal mycelium compared with the control when using *Cchp11* and *CcUP18* as reference genes (Figures 6E,F).

These results indicated that (i) the use of unstable reference genes will lead to differences in the relative transcript profile and (ii) the use of different suitable references could have diverse significant results during qPCR.

## Discussion

To analyze the gene functions of the tea plant pathogen *C. camelliae*, gene expression differences might be important to analyze (Lu et al., 2018). RT-qPCR has become an important technique for studying gene transcript profiles as its sensitivity, accuracy, and reproducibility (Klein, 2002; Bustin et al., 2005; Hao et al., 2014; Wang et al., 2017). The reliability of the results of gene expression in RT-qPCR studies is dependent on the use of suitable reference genes for the microbe and the condition under study (Galli et al., 2015; Huang et al., 2018). The expression of the reference genes should not change with time or under different experimental conditions (Bustin, 2002; Melgar-Rojas Pedro et al., 2015). However, the stability among traditionally used reference genes is relative, and there is no single gene that has a constant stable expression under all experimental conditions (Radonic et al., 2004; Czechowski et al., 2005). Under specific experimental conditions, it might be misleading to use previously identified reference genes for the normalization of target gene expression in *C. camelliae* without first investigating their stability (Amil-Ruiz et al., 2013; Galli et al., 2015). Therefore, *CcSPAC6B12.04c*, *CcWDR83*, *Cchp11*, *Ccnew1*, *CcRNF5*, *CcHpcob*, *CcfaeB-2*, *CcYER010C*, *CcRNM1*, *CcUP18*, and *CcACT* were selected here for validation under the experimental conditions.

The expression of candidate reference genes were first evaluated with the Cq value in RT-qPCR. The Cq values for most of the tested samples were approximately 25. Even after 1000-fold dilution, the Cq value for nine candidate genes was still lower than 35.0. All candidate reference genes had very good linear amplification, and four of them had *R*^2^ values greater than 99%. Notably, the *R*^2^ value of *CcSPAC6B12.04* was 100%. The Cq value comparison provided an approximation of the stability of gene expression.

The programs geNorm, NormFinder, and Bestkeeper were then used to determine which reference gene was most suitable for transcript normalization during *C. camelliae* spore germination, mycelial growth, and fungal interaction with the tea plants. Among the 12 candidate reference genes, *Ccnew1* was ranked first in both the geNorm and NormFinder analyses under all conditions. The Cq SD value of *Ccnew1* was < 1 based on the Bestkeeper program, which was consistent with reference genes with SD values < 1 that are considered stable (Pfaffl et al., 2004; Marcial-Quino et al., 2016). Taken together, *Ccnew1* was the most stable reference gene for the detection of target gene expression not only during *C. camelliae* spore germination and its interaction with hosts, but also during mycelial growth. Here, the result indicates that *Ccnew1* is a universal reference gene that is stably expressed under different experimental conditions in this study.

We further used Refinder analysis to reduce bias or avoid contradictory results caused by the use of individual methods, ΔCt, Bestkeeper, geNorm, and NormFinder (Xie et al., 2011; Marcial-Quino et al., 2016). Based on this, the most stable reference genes were *Ccnew1*, *CcHplo* and *CcSPAC6B12.04c*, while the least stable genes were *Cchp11* and *CcUP18.* Interestingly, *CcfaeB-2* was ranked the second most stable gene not only in *C. camelliae* spore germination but also in mycelial growth (Figures 4A,B), whereas it ranked the second least stable reference gene under all conditions (Figure 4C). One reason that explains the difference may be the highest variation in expression of *CcfaeB-2* (Figure 3).

During previous studies of gene expression in *Colletotrichum* spp., *ACT* was often used to normalize qPCR because it was stably expressed in many other microbes (Narusaka et al., 2009; Liu et al., 2017). Similarly, *CcACT* seems stably expressed during *C. camelliae* mycelial growth (Figure 4B). However, under the combined conditions, *CcACT* was ranked as the least stably expressed reference gene (Figure 4C). Nevertheless, if only the expression of target genes was detected during mycelial growth, then *CcACT* could be a choice for reference gene (Figure 6D). In conclusion, the commonly used reference genes need to be reconfirmed according to specific experimental conditions.

To validate the suitability of potential reference genes, the expression profile of a target gene was assessed in *C. camelliae*, with *Cenew1*, *CcHplo*, *CcSPAC6B12.04c*, *CcACT*, *Cchp11*, and *CcUP18* as internal reference genes. The gene expression patterns were highly similar but the expression levels were significantly different from that of the treatments when the most stably expressed reference genes were used, while the transcript levels could be inaccurate or present no significant differences when the least stably expressed reference genes were used (Figures 5, 6). Thus, using a reliable reference gene is a prerequisite for accurate RT-qPCR data analyses of *C. camelliae*.

## Conclusion

To our knowledge, this is the first report describing the identification of suitable reference genes for RT-qPCR analyses in *C. camelliae*. We evaluated 12 candidate reference genes for the normalization of gene expression in *C. camelliae*. Common statistical algorithms and a web-based analysis program were used and indicated that *Cenew1*, *CcHplo*, and *CcSPAC6B12.04c* were the most stable reference genes. In addition, *Cchp11* and *CcUP18* seem to be unsuitable as internal controls under the experimental conditions we tested. Additionally, the analysis of the *CcABC8* expression level confirmed the importance of selecting suitable reference genes for the normalization of RT-qPCR data. The reference genes selected here provide important choices for target gene expression and functional studies in *C. camelliae*.

## Data Availability

All datasets generated for this study are included in the manuscript and/or the Supplementary Files.

## Author Contributions

SL designed the experiments. SH, TA, and RA performed the experiments. SL, SH, and RA analyzed the data. SH and SL wrote the manuscript.

## Conflict of Interest Statement

The authors declare that the research was conducted in the absence of any commercial or financial relationships that could be construed as a potential conflict of interest.

## Footnotes

1 https://blast.ncbi.nlm.nih.gov/Blast.cgi

2 https://www.ncbi.nlm.nih.gov/tools/primer-blast/
