# Supplementary material for: Validation of Reliable Reference Genes for RT-qPCR Studies of Target Gene Expression in Colletotrichum camelliae During Spore Germination and Mycelial Growth and Interaction With Host Plants
Source: Front Microbiol. 2019 Sep 4;10:2055. doi: 10.3389/fmicb.2019.02055 (PMC6737088; doi:10.3389/fmicb.2019.02055)
Supplement: Supplementary file 1 [file Data_Sheet_1.docx]

| **Gene** | **Treatment T1** | | | **Treatment T2** | | | **Treatment T3** | | |
| --- | --- | --- | --- | --- | --- | --- | --- | --- | --- |
|  | **Repl_1** | **Repl_2** | **Repl_3** | **Repl_1** | **Repl_2** | **Repl_3** | **Repl_1** | **Repl_2** | **Repl_3** |
| CcRNM1 | 1081.0 | 1003.0 | 810.0 | 810.0 | 1007.0 | 866.0 | 1103.0 | 860.0 | 1146.0 |
| CcSPAC6B12.04c | 594.0 | 623.0 | 410.0 | 423.0 | 526.0 | 498.0 | 604.0 | 478.0 | 539.0 |
| CcHpcob | 2730.0 | 3197.0 | 3151.0 | 2051.0 | 3236.0 | 3359.0 | 3613.0 | 2800.0 | 2850.0 |
| Ccnew1 | 274.0 | 230.0 | 157.0 | 209.0 | 253.0 | 222.0 | 312.0 | 247.0 | 311.0 |
| CcHplo | 1356.0 | 1252.0 | 841.0 | 961.0 | 1230.0 | 1083.0 | 1228.0 | 1001.0 | 1210.0 |
| CcfaeB-2 | 64.0 | 77.0 | 38.0 | 49.0 | 64.0 | 46.0 | 56.0 | 42.0 | 77.0 |
| CcWDR83 | 279.0 | 273.0 | 230.0 | 217.0 | 280.2 | 209.0 | 249.0 | 177.0 | 301.0 |
| Cchp11 | 190.0 | 208.0 | 127.0 | 114.0 | 174.0 | 185.0 | 195.0 | 168.0 | 157.0 |
| CcRNF5 | 528.0 | 568.0 | 451.0 | 394.0 | 565.0 | 422.0 | 540.0 | 452.0 | 525.0 |
| CcYER010C | 68.0 | 69.0 | 56.0 | 23.0 | 90.0 | 83.0 | 73.0 | 62.0 | 61.0 |
| CcUP18 | 20.0 | 32.0 | 34.0 | 25.0 | 33.0 | 22.0 | 38.0 | 18.0 | 32.0 |

**Table S1.** The counts of candidate reference genes in *C. camelliae* during interaction with hosts at different time points observed by the RNA sequence.

| **Reference**  **gene** | **Total amino acid** | **MW （kDa）** | **PI** | **Homologous**  **Specie** | **Colletotrichum Ortholog** | **Similarity**  **(%)** |
| --- | --- | --- | --- | --- | --- | --- |
| CcUP18 | 217 | 26.3 | 11.0 | *C.fructicola* Nara gc5 | ELA35911 | 89.5 |
| CcSPAC6B12.04c | 452 | 50.4 | 6.2 | *C.fructicola* Nara gc5 | ELA24928 | 98.9 |
| CcRNM1 | 194 | 21.6 | 4.3 | *C.gloeosporioides* Cg-14 | EQB58236 | 98.97 |
| CcHpcob | 876 | 100.5 | 5.9 | *C.gloeosporioides* Cg-14 | EQB50494 | 98.4 |
| Ccnew1 | 78 | 8.6 | 10.4 | *C.gloeosporioides* Cg-14 | EQB44976 | 98.7 |
| CcHplo | 404 | 44.8 | 6.9 | *C.gloeosporioides* Cg-14 | EQB45128 | 92.2 |
| CcfaeB-2 | 559 | 60.5 | 4.7 | *C.gloeosporioides* Cg-14 | EQB48532 | 95.0 |
| CcWDR83 | 361 | 38.6 | 7.6 | *C.gloeosporioides* Cg-14 | EQB49172 | 97.0 |
| Cchp11 | 572 | 64.3 | 5.4 | *C.gloeosporioides* Cg-14 | EQB55695 | 96.9 |
| CcRNF5 | 405 | 43.3 | 4.7 | *C.gloeosporioides* Cg-14 | EQB48134 | 82.3 |
| CcYER010C | 218 | 23.6 | 7.2 | *C.gloeosporioides* Cg-14 | EQB57386 | 98.6 |

**Table S2.** Bioinformatics analysis of the candidate reference genes.

A.


B.

C.

D.

F.

G.

H.

I.

J.

K.

**Figure S1.** The alignment of candidate reference genes encoding proteins with their orthologous proteins analyzed by DNAMAN. A-K, The alignment of CcRNM1, CcSPAC6B12.04c, CcHpcob, Ccnew1, CcHplo, CcfaeB-2, CcWDR83, Cchp11, CcRNF5, CcYER010C, and CcUP18 with the orthologous protein, respectively.
